# Honokiol alleviates sepsis-induced acute kidney injury in mice by targeting the miR-218-5p/heme oxygenase-1 signaling pathway

**DOI:** 10.1186/s11658-019-0142-4

**Published:** 2019-02-22

**Authors:** Tao Zhang, Lei Xiang

**Affiliations:** 1 Department of of Intensive Care Unit, Tianjin Huanhu Hospital, No. 6 Jizhao Road, Tianjin, 300060 People’s Republic of China; 2 Department of Neurology, Tianjin Huanhu Hospital, Tianjin, 300060 People’s Republic of China

**Keywords:** Honokiol, Sepsis, Acute kidney injury, Heme oxygenase-1

## Abstract

**Background:**

Honokiol is a low-molecular-weight natural product and has been reported to exhibit anti-inflammatory activity.

**Objectives:**

Our study aimed to investigate the influence of honokiol on sepsis-induced acute kidney injury (AKI) in a mouse model.

**Material and methods:**

A cecal ligation and puncture (CLP) surgical operation was performed to establish a sepsis-induced acute kidney injury model in mice. Renal histomorphological analysis was performed with periodic acid-Schiff (PAS) staining. The levels of inflammatory markers in serum were measured by ELISA assay. The mRNA and protein levels were assayed by RT-qPCR and western blotting, respectively. Annexin V-FITC/PI staining was used to evaluate glomerular mesangial cell (GMC) apoptosis.

**Results:**

The results revealed that honokiol significantly increased the survival rate in mice undergoing a CLP operation. Inflammatory cytokines, such as TNF-α, IL-6 and IL-1β, were significantly inhibited in honokiol-treated septic mice compared with the CLP group. In addition, honokiol showed the ability to reverse CLP-induced AKI in septic mice. Furthermore, heme oxygenase-1 (HO-1) expression levels were significantly up-regulated and miR-218-5p was markedly down-regulated in honokiol-treated septic mice as compared to CLP-operated mice. Bioinformatics and experimental measurements showed that HO-1 was a direct target of miR-218-5p. In vitro experiments showed that both honokiol and miR-218-5p inhibitors blocked lipopolysaccharide (LPS)-induced cell growth inhibition and GMC apoptosis by increasing the expression of HO-1.

**Conclusions:**

Honokiol ameliorated AKI in septic mice and LPS-induced GMC dysfunction, and the underlying mechanism was mediated, at least partially, through the regulation of miR-218-5p/HO-1 signaling.

**Electronic supplementary material:**

The online version of this article (10.1186/s11658-019-0142-4) contains supplementary material, which is available to authorized users.

## Introduction

Sepsis is caused by infections with bacteria such as *Staphylococcus aureus* and is characterized by a whole-body inflammatory response, which is a leading cause of death in intensive care units (ICUs) [1]. There have been an increasing number of studies showing that acute kidney injury (AKI) is a frequent and serious complication of sepsis in ICU patients, accounting for 50% or more of cases of AKI in ICUs, and is associated with a very high mortality [2]. In clinical practice, there are approximately 1000,000 reported cases and more than 160,000 deaths each year attributable to sepsis in the United States alone [3]. Although inflammatory reaction triggered by cytokine production is a leading cause of sepsis-induced multiple organ system failure, little progress has been made in the management of sepsis. Therefore, it is important to explore a novel and effective adjuvant therapy drug for sepsis-induced organ failure.

Honokiol is a low-molecular-weight natural product isolated and purified from *Magnolia officinalis*, which has been shown to participate in a variety of physiological and pathological processes [4, 5]. Previous studies have demonstrated that honokiol alleviates experimental mesangial proliferative glomerulonephritis [6] and inhibits LPS- and high-glucose-induced upregulation of inflammatory cytokines in human renal mesangial cells (HRMCs) [7, 8]. Moreover, honokiol ameliorates renal fibrosis by inhibiting extracellular matrix and pro-inflammatory factors in vivo and in vitro [9]. Honokiol protects against renal ischemia/reperfusion injury via the suppression of oxidative stress and inflammatory reactions in a rat model [10]. Interestingly, honokiol ameliorates sepsis-associated acute lung injury [5] and kidney injury [11] in murine models via the inhibition of oxidative stress and inflammation. However, the underlying molecular mechanisms of sepsis-induced AKI and the protective effect of honokiol in sepsis-induced organ failure have not been clearly delineated.

Heme oxygenase-1 (HO-1) as an inducible enzyme plays a crucial role in anti-oxidation, anti-inflammation, anti-apoptosis and cytoprotection [12]. Extensive evidence has demonstrated that HO-1 has a protective effect against AKI in various animal models, such as sepsis-, ischemia-reperfusion- and kidney transplantation-induced AKI [13–15]. HO-1 knockout exhibits enhancement of the inflammatory response and structural renal injury [16, 17]. In human renal epithelial cells, overexpression of HO-1 inhibits reactive oxygen species generation, autophagy and apoptosis and promotes cell survival during hypoxia and oxidative stress [18, 19].

MicroRNAs (miRNAs) have been revealed as small, single-stranded and non-coding RNAs that contribute to post-transcriptional regulatory mechanisms and regulate gene expression by binding to the 3′-untranslated regions (3′-UTRs) [20]. MiRNAs have been shown to be involved in a variety of biological processes, including sepsis-induced AKI. For example, miR-107, miR-124, miR-204 and miR-590 are mediators of sepsis-induced AKI for their ability to specifically bind to 3′-UTRs to inhibit translation of the target genes [21, 22]. In the present study, we aimed to investigate the role of miR-218-5p and its target gene HO-1 in sepsis-induced AKI and hypothesized that honokiol could ameliorate sepsis-induced AKI by targeting the miR-218-5p/HO-1 signaling pathway. This study will further increase our understanding of the renoprotective effect of honokiol, which may be useful in managing sepsis-induced renal failure.

## Materials and methods

### Experimental animals

Ten-week-old male ICR mice (*n* = 80; body weight: 20–25 g) were obtained from Vital River Laboratories Co., Ltd. (Beijing, China) and allowed to acclimatize to the environment for 1 week. The mice were fed under controlled conditions – temperature 25 ± 2 °C, humidity 55 ± 5% and 12-h light/dark cycle – and the mice were given free access to food and tap water. Sepsis was induced in mice undergoing cecal ligation and puncture (CLP) surgery as previously described [23]. Honokiol (Aldrich Chemical Co., Milwaukee, WI, USA; high performance liquid chromatography, 98%; formula: C_18_H_18_O_2_) dissolved in dimethyl sulfoxide (DMSO) was intraperitoneally injected into CLP-induced septic mice at the concentration of 2.5 mg/kg (low dose, *n* = 10) or 10 mg/kg (high dose, n = 10) body weight after CLP treatment twice within 24 h. The mice in the control group were intraperitoneally injected with 2 ml of PBS. In another experiment, the 96 h survival of CLP mice with or without honokiol treatment was observed (*n* = 10 in each group). The study was approved by the Ethics Committee of Tianjin Huanhu Hospital, Tianjin, China.

### Bacterial counts in blood and organs

Twenty-four hours after the CLP operation, the mice were killed, and the blood, kidney, liver and brain were collected and homogenized in 2 ml of sterile PBS. After serial dilution, the homogenates and the blood were spread on LB agar plates and incubated at 37 °C for 24 h. Colonies were counted and expressed as CFU/organ.

### Serum inflammatory cytokines

Twenty-four hours after CLP operation, blood was collected and centrifuged, and the levels of tumor necrosis factor-α (TNF-α; cat. no. E-EL-M0049), interleukin (IL)-1β (cat. no. E-EL-M0037) and IL-6 (cat. no. E-EL-M0044) in serum were detected using a mouse bioactive ELISA assay, according to the manufacturer’s protocol (Elabscience Biotechnology Co., Ltd., Wuhan, China).

### Histomorphology and immunohistochemistry

Formalin-fixed and paraffin-embedded kidney tissues were cut into about 4 μm-thick sections, which were stained with periodic acid-Schiff (PAS) staining (Abcam, Cambridge, UK), and visualized under a microscope (Leica DM 2500; Leica Microsystems GmbH, Wetzlar, Germany).

### Cell culture

Mouse glomerular mesangial cells (GMCs; serial number: 3131C0001000300021) were obtained from the Institute of Biochemistry and Cell Biology of the Chinese Academy of Sciences (Shanghai, China). Cells were cultured in Dulbecco’s modified Eagle’s medium-F12 (DMEM-F12; Gibco; Thermo Fisher Scientific, Inc., Waltham, MA, USA) with 5% fetal bovine serum (Beyotime Institute of Biotechnology, Haimen, China), 2 mM _L_-glutamine, 100 U/ml penicillin and 100 mg/ml streptomycin in a humidified incubator (Thermo Fisher Scientific, Inc.), 5% CO_2_, 95% air atmosphere. Lipopolysaccharide (LPS; 10 μg/mL; Sigma-Aldrich, Merck KgaA, Germany) was exposed to GMCs with or without honokiol treatment at various concentrations (0, 1, 10, 100 and 200 μM).

### Luciferase reporter assay

The conserved binding sites between miR-218-5p and HO-1 were obtained using the online predict software TargetScan (http://www.targetscan.org) and miRDB (http://www.mirdb.org/miRDB/). miR-Con, miR-218-5p mimics and inhibitors were synthesized by RiboBio (Guangzhou, China). GMCs were seeded in 6-well plates and transfected with miR-Con, miR-218-5p mimics and inhibitors using Lipofectamine 2000 (Invitrogen; Thermo Fisher Scientific, Inc., Waltham, MA, USA) for 48 h at 37 °C according to the manufacturer’s protocol. The wild-type (WT) and mutant-type (MT) 3′-UTR of HO-1 were inserted into the multiple cloning sites of the luciferase expressing pMIR-REPORT vector (Ambion; Thermo Fisher Scientific, Inc.). For the luciferase assay, GMCs (1 × 10^5^) were seeded into 24-well plates and co-transfected with luciferase reporter vectors containing the WT or MT 3′-UTR of HO-1 (0.5 μg) and mimic sequences of miR-218-5p (100 nM) using Lipofectamine 2000 (Invitrogen; Thermo Fisher Scientific, Inc.). The luciferase activity was measured using a luciferase reporter assay kit (Promega, Madison, WI, USA) according to the manufacturer’s protocol.

### Cell viability

After treatment with different conditions for 24, 48 or 72 h, cell viability was measured using the CCK-8 Cell Proliferation/Viability Assay Kit (Dojindo, Japan). Absorbance was recorded at 450 nm using an Elx800 Reader (Bio-Tek Instruments Inc., Winooski, VT, USA).

### Cell apoptosis

After treatment with different conditions for 48 h, an Annexin V-FITC/PI kit (Becton, Dickinson and Company, New Jersey, USA) was used to stain cells for 15 min, and then a cell apoptosis assay was performed by flow cytometry assay (FACScan, BD Biosciences, San Jose, CA, USA) and analyzed by CELL Quest 3.0 software (BD Biosciences).

### Reverse transcription-quantitative polymerase chain reaction (RT-qPCR)

#### miRNA RT-qPCR

Total RNA was extracted from kidney using TRIzol (Invitrogen; Thermo Fisher Scientific, Inc., USA). RT was performed using a TaqMan reverse transcription kit (Applied Biosystems; Thermo Fisher Scientific, Inc.), according to the manufacturer’s protocol. MiRNAs were detected using a TaqMan MicroRNA assay (Applied Biosystems; Thermo Fisher Scientific, Inc.), according to the manufacturer’s protocol. U6 small nuclear RNA was used as an endogenous control. Relative miRNA expression levels were calculated using the 2^-ΔΔCq^ method [24]. The primers of miRNAs are shown in Additional file 1: Table S1.

#### mRNA RT-qPCR

The cDNA was synthesized by reverse transcription reactions with 2 μg of total RNA using Moloney murine leukemia virus reverse transcriptase (Invitrogen; Thermo Fisher Scientific, Inc.) according to the manufacturer’s protocol. RT-qPCR was performed by the Applied Biosystems 7300 Real-Time PCR System (Thermo Fisher Scientific, Inc.) with the TaqMan Universal PCR Master Mix (Thermo Fisher Scientific, Inc.). Glyceraldehyde 3-phosphate dehydrogenase (GAPDH) served as an internal standard. Relative gene expression was calculated using the 2^−ΔΔCq^ method [24]. PCR was performed with the following primers: Bcl-2, forward 5′-CCAGCGTGTGTGTGCAAGTGTAAAT-3′ and reverse 5′-ATGTCAATCCGTAGGAATCCCAACC-3′; BAX, forward 5′-CAGGGTTTCATCCAGGATCGAGCAGG-3′ and reverse 5′-CGGGGGGAGTCCGTGTCCACGTCAG-3′. HO-1, forward 5′-CAGCCCCACCAAGTTCAAAC-3′ and reverse 5′-GTCTTTGTGTTCCTCCTCTGTCAGCAT-3′; GAPDH, forward 5′-GCACCGTCAAGCTGAGAAC-3′ and reverse 5′-TGGTGAAGACGCCAGTGGA-3′.

### Western blotting

Protein was extracted from kidneys using NP-40 buffer (Thermo Fisher Scientific, Inc.), followed by 5–10 min boiling and centrifugation at 10,000 g, for 10 min at 4 °C to obtain the supernatant and was quantified using the Bicinchoninic Acid kit for Protein Determination (no. BCA1-1KT; Sigma-Aldrich; Merck Millipore). Samples containing 30 μg of proteins were separated by 10% SDS-PAGE and transferred to nitrocellulose membranes (Bio-Rad Laboratories, Inc., Hercules, CA, USA). Membranes were blocked for 2 h at room temperature with 5% (*w*/*v*) non-fat dry milk in tris-buffered saline and 0.1% (w/v) Tween 20 (TBST), and then incubated with primary antibodies specific for HO-1 (cat. no. sc-1796, 1: 1000; Santa Cruz Biotechnology, Inc., Dallas, TX, USA). β-actin (cat. no. sc-81,178; dilution, 1: 2000; Santa Cruz Biotechnology) was used as a protein loading control. The membranes were next incubated with the appropriate HRP (horseradish peroxidase)-conjugated antibody (cat. no. sc-516,102; 1:10,000; Santa Cruz Biotechnology) at room temperature for 2 h. Signal detection was carried out with an enhanced chemiluminescence system (GE Healthcare, Chicago, IL, USA), and protein bands were analyzed with Quantity One software version 4.5 (Bio-Rad Laboratories, Inc., Hercules, CA, USA).

### Statistical analysis

The data from these experiments were reported as mean ± SEM for each group. All statistical analyses were performed using PRISM version 7.0 (GraphPad Software, Inc., La Jolla, CA, USA). Student’s *t*-test was used to analyze two-group differences. Inter-group differences were analyzed by one-way analysis of variance, followed by a post hoc Tukey test for multiple comparisons. A *P* value less than 0.05 was considered to indicate a statistically significant difference.

## Results

### Honokiol improves survival in mice after *S. aureus* or CLP treatment

**Fig. 1:**
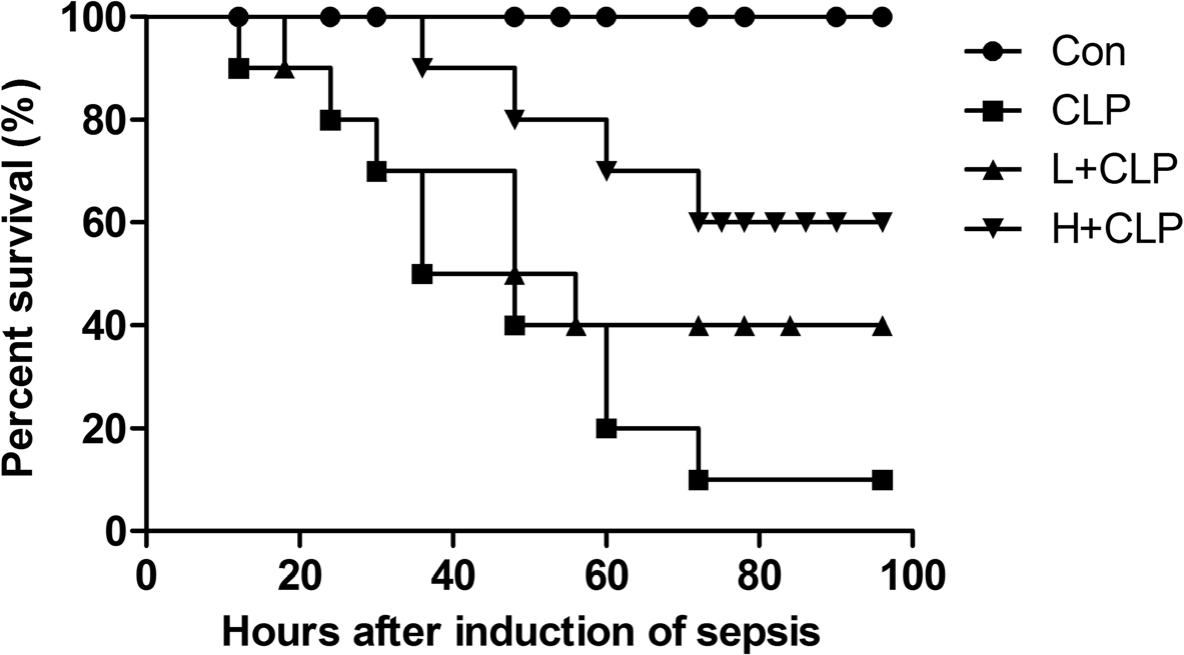
Survival curves of mice in CLP-induced sepsis with or without honokiol treatment

To determine the functional roles of honokiol in sepsis in mice, honokiol (2.5 mg/kg or 10 mg/kg) was administered to mice 30 min after CLP treatment. The survival of these mice was monitored for 4 days after the induction of sepsis by the CLP operation. The results demonstrated that both low-concentration and high-concentration honokiol significantly increased the survival in mice undergoing CLP as compared to mice only treated with CLP (Fig. 1). Sepsis was induced in mice by CLP; the survival rates in CLP, L + CLP and H + CLP groups were 10, 40, and 60%, respectively, after 4 days of treatment (Fig. 1).

### Bacterial counts in septic mouse organs are inhibited after honokiol treatment

**Fig. 2:**
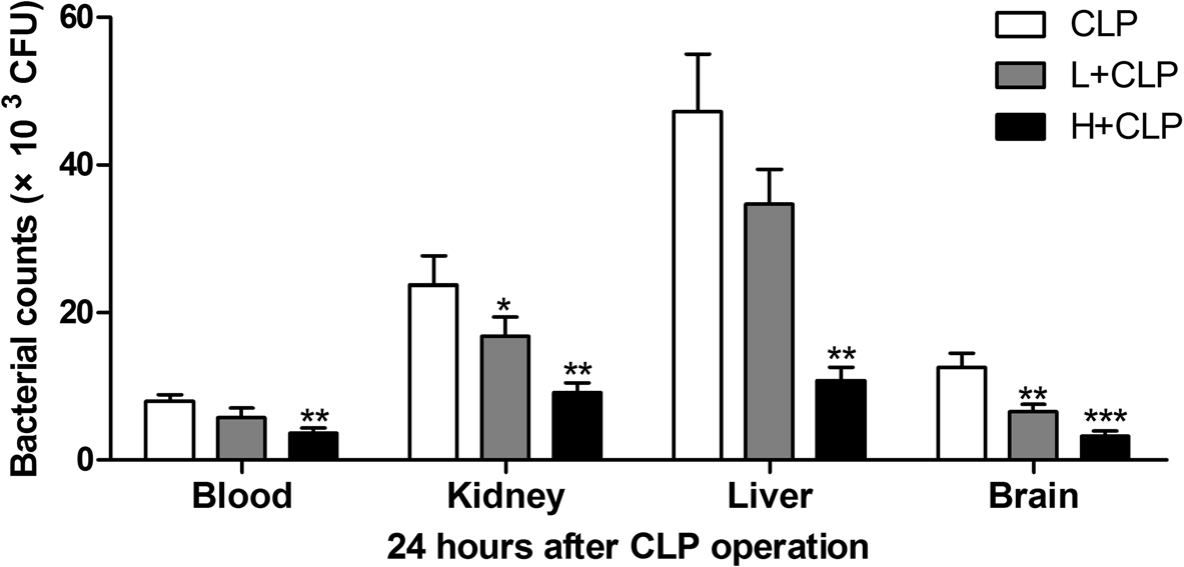
The blood, kidney, liver and brain were collected, and bacterial counts were measured in CLP-induced septic mice. Values are expressed as mean ± SEM, *n* = 10 in H + CLP group and *n* = 8 in CLP or L + CLP group. ^*^ *P* < 0.05, ^**^ *P* < 0.01, ^***^ *P* < 0.001, versus *S. aureus* or CLP single treatment group

Bacterial counts in blood, kidney, liver and brain were measured after induction of sepsis with CLP treatment for 24 h. The bacterial counts in blood, kidney, liver and brain were significantly higher in the CLP (Fig. 2) group than that of honokiol administration groups. These data suggest that honokiol exhibits strong bacteria-fighting ability in septic mice.

### Honokiol inhibits serum inflammatory cytokines in septic mice

**Fig. 3:**
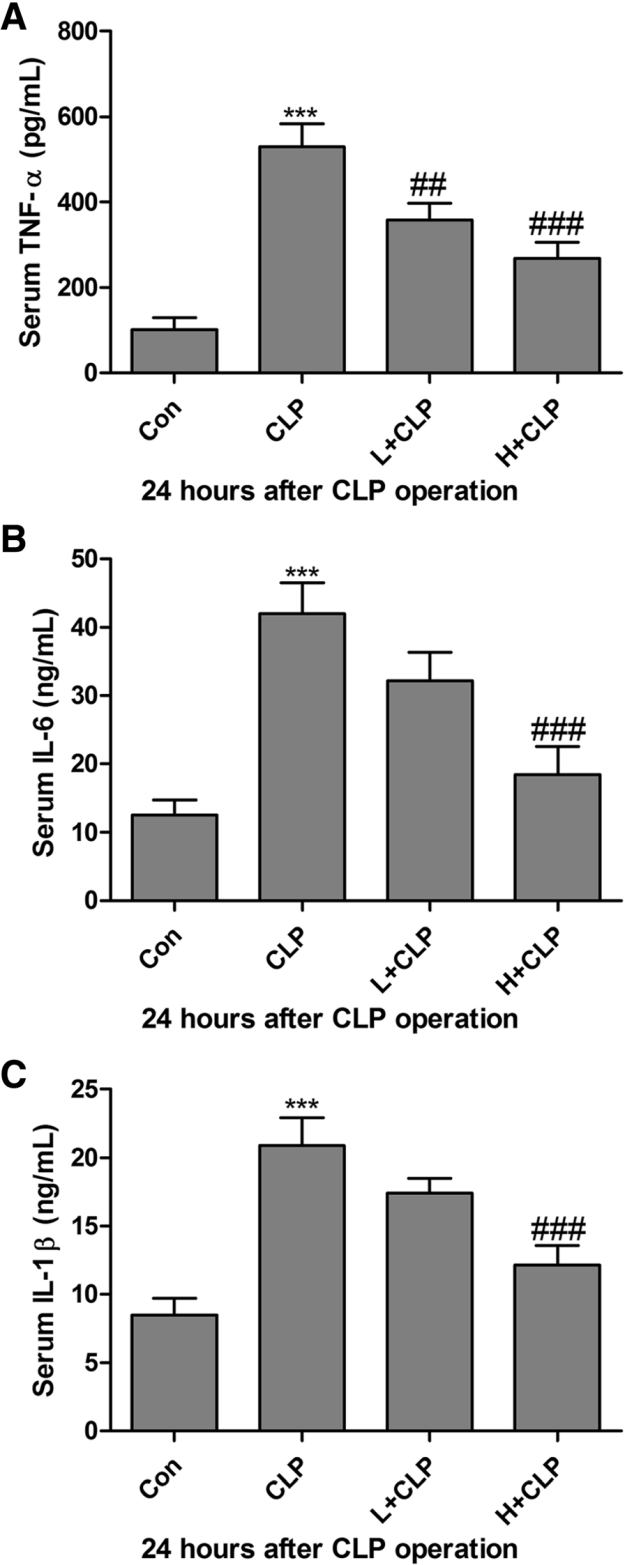
Honokiol inhibits serum inflammatory cytokines in septic mice. Serum levels of TNF-α (**a**), IL-6 (**b**) and IL-1β (**c**) were detected using mouse bioactive ELISA assay in CLP-induced septic mice. Values are expressed as mean ± SEM, n = 10 in Con or H + CLP group and n = 8 in CLP or L + CLP group. ^***^ *P* < 0.001, versus control group; ^##^ *P* < 0.01 and ^###^ *P* < 0.001 versus CLP single treatment group

A dramatic increase in inflammatory cytokine levels is one of the major clinical features of sepsis [25]. In the present study, serum levels of TNF-α, IL-6 and IL-1β in septic mice and honokiol-treated septic mice were measured. As shown in Fig. 3a, serum levels of TNF-α were significantly increased in the CLP group as compared to the corresponding control group. However, honokiol administration markedly reversed CLP-induced up-regulation of TNF-α in septic mice. In addition, compared with the control group, the serum levels of IL-6 and IL-1β were markedly induced in the CLP-induced (Fig. 3b and c) septic mice model. However, honokiol administration significantly reduced IL-6 and IL-1β in septic mice.

### Honokiol ameliorates AKI in septic mice by targeting HO-1

**Fig. 4:**
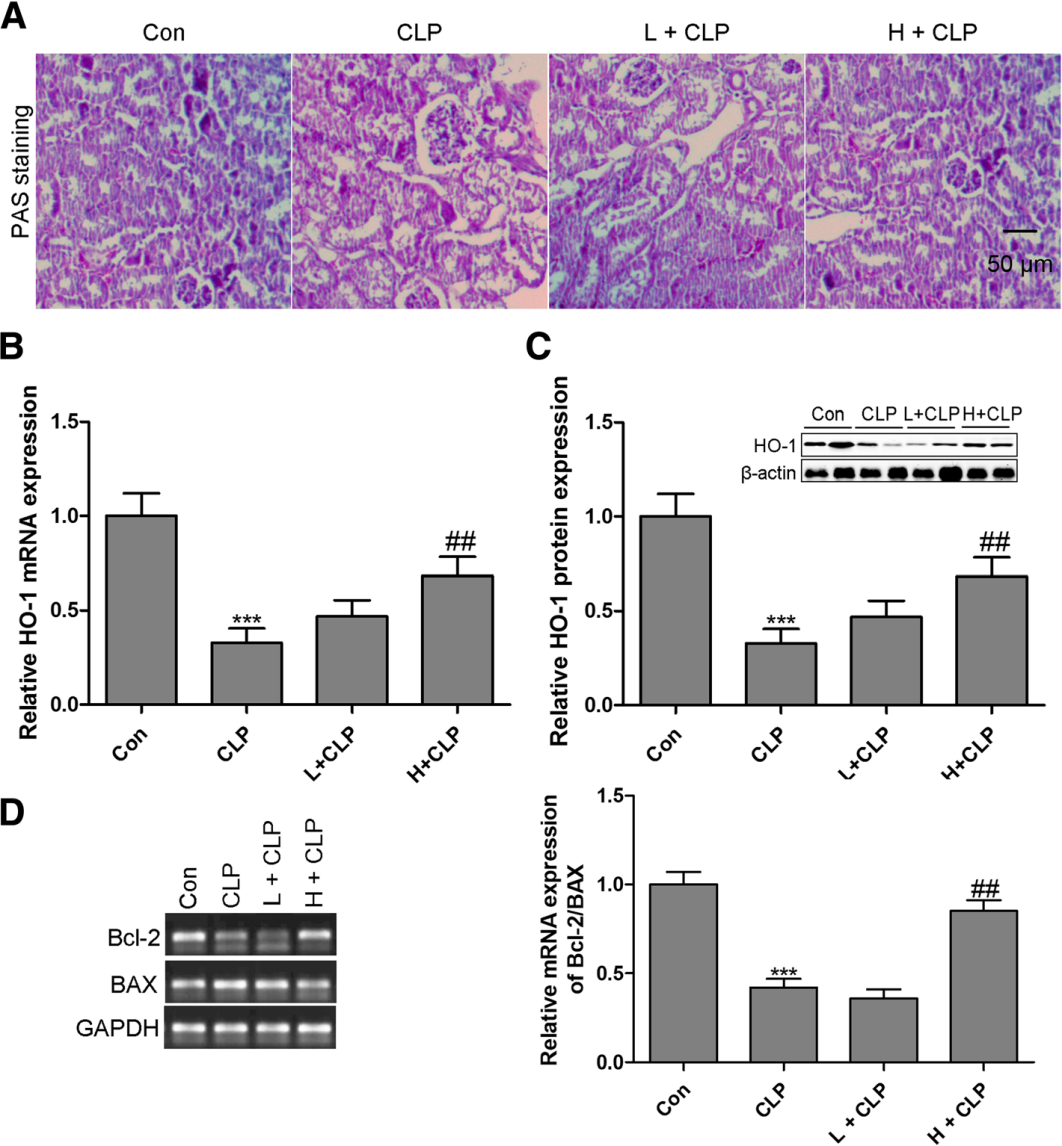
Honokiol ameliorates AKI in septic mice by targeting HO-1. Renal pathology PAS staining (× 200) was performed in CLP-induced septic mice for 24 h (**a**). The mRNA (**b**) and protein (**c**) expression levels of HO-1 were measured by RT-qPCR and western blotting, respectively, in the kidney of septic mice. The mRNA expression levels of Bcl-2 and BAX were measured by RT-qPCR in the kidney of septic mice (**d**). Values are expressed as mean ± SEM, n = 10 in Con or H + CLP group and *n* = 8 in CLP or L + CLP group. ^***^ *P* < 0.001, versus control group; ^#^ *P* < 0.05, ^##^ *P* < 0.01, ^###^ *P* < 0.001, versus CLP single treatment group

To determine the role of honokiol in CLP-induced AKI in the septic mouse model, renal histological examination by PAS was implemented. The results indicated that a relatively intact structure of the kidney tissues was observed in normal mice. However, the CLP group showed significant glomerular and tubulointerstitial damage, which was restored in the presence of honokiol (Fig. 4a). These results show that honokiol significantly improves renal physiological structure and function in septic mice. To determine the effect of honokiol on HO-1 expression in CLP-induced AKI, the mRNA and protein expression levels of HO-1 were measured in the kidney of honokiol-treated septic mice. As shown in Fig. 4b and c, the levels of HO-1 mRNA and protein in the kidney were much lower in the CLP model group as compared to those in the control group. In contrast, the mRNA and protein expression levels of HO-1 in the kidney of septic mice were dramatically up-regulated with honokiol treatment. We also found that the apoptosis-related signaling pathway was activated in the kidney of septic mice, reflecting that the ratio of Bcl-2 to BAX mRNA expression level was down-regulated in the kidney of septic mice. However, CLP-induced apoptosis in the kidney was attenuated by honokiol treatment with high concentration (Fig. 4d).

### MiRNA expression in the kidney from septic mice

**Fig. 5:**
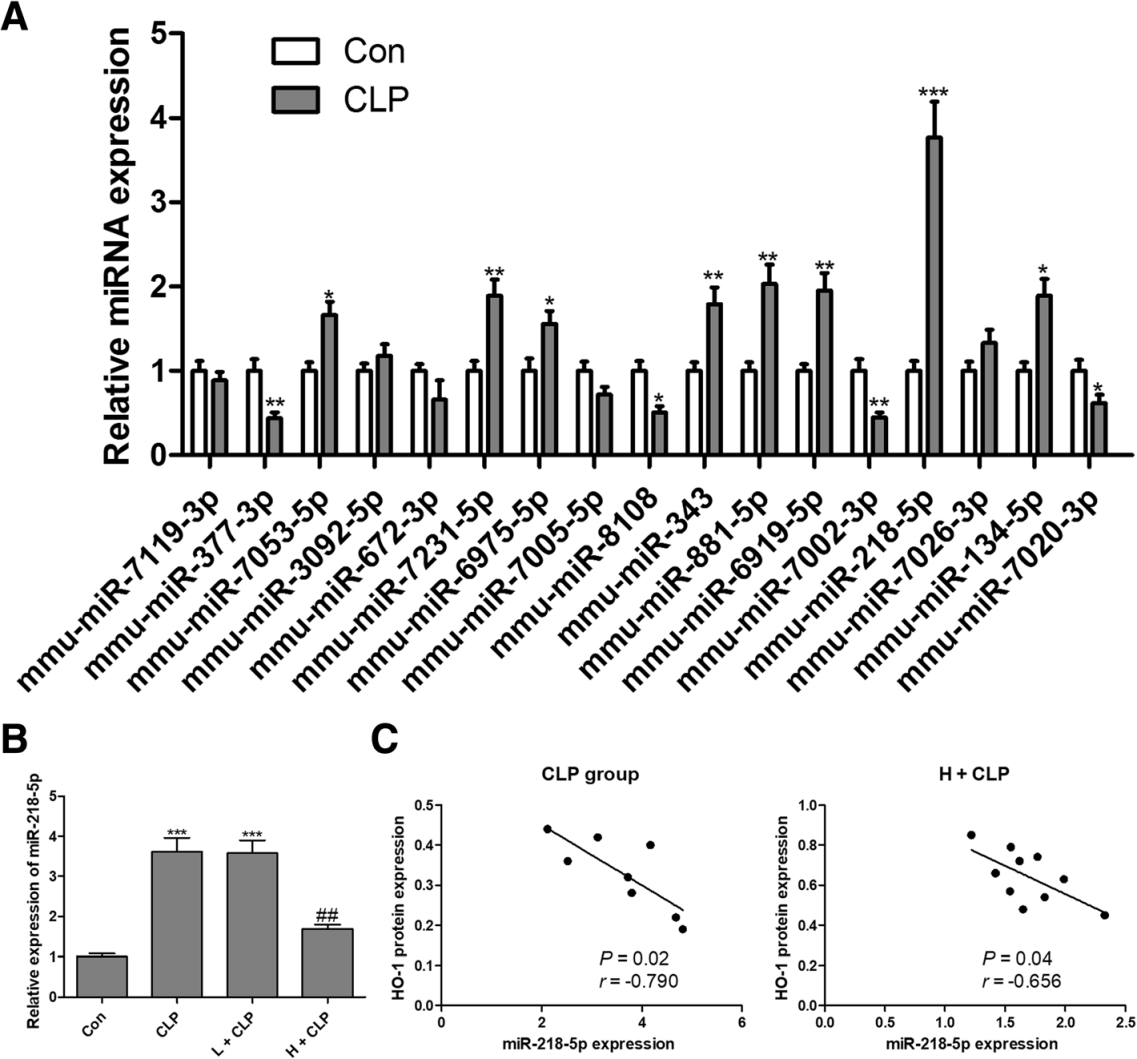
MiRNA expression in the kidney from septic mice. HO-1-related miRNAs were predicted using online software TargetScan (www.targetscan.org) and miRDB (http://www.mirdb.org/miRDB/), and the expression levels of 17 miRNAs were measured by RT-qPCR assays (**a**). After treatment with honokiol, the expression of miR-218-5p was measured using RT-qPCR in the kidney (**b**). Spearman’s rank analysis was used to identify the correlation between HO-1 and miR-218-5p expression levels in the kidney of septic mice with CLP or CLP combined with honokiol treatment (**c**). Values are expressed as mean ± SEM, *n* = 10 in Con or H + CLP group and n = 8 in CLP or L + CLP group. ^*^ *P* < 0.05, ^**^ *P* < 0.01, ^***^ *P* < 0.001, versus control group; ^##^ *P* < 0.01 versus CLP group

To determine whether HO-1-related miRNAs were involved in sepsis-induced AKI, we used the online prediction software TargetScan (www.targetscan.org) and miRDB (http://www.mirdb.org/miRDB/) to identify potential miRNAs that could target HO-1. Using these approaches, 17 miRNAs (miR-7119-3p, miR-377-3p, miR-7053-5p, miR-3092-5p, miR-672-3p, miR-7231-5p, miR-6975-5p, miR-7005-5p, miR-8108, miR-343, miR-881-5p, miR-6919-5p, miR-7002-3p, miR-218-5p, miR-7026-3p, miR-134-5p and miR-7020-3p) were identified as candidate miRNAs. Among these miRNAs, 8 miRNAs were significantly up-regulated and 4 miRNAs were significantly down-regulated in the kidney from septic mice compared with that of the control group (Fig. 5a). In addition, the expression of 5 miRNAs showed no significant difference between the two groups (Fig. 5a). We found that the fold change of miR-218-5p was approximately 3.8-fold at the highest level. Therefore, we selected out miR-218-5p for further investigation. Intriguingly, CLP-induced up-regulation of miR-218-5p in the kidney of septic mice was significantly reduced by honokiol with high concentration (Fig. 5b). We also found a significant negative correlation between miR-218-5p and HO-1 protein expression in both septic mice (Fig. 5c) and septic mice with honokiol treatment at high concentration (Fig. 5c). These findings suggested that HO-1 and miR-218-5p might play reciprocal roles in the progression of CLP-induced AKI.

### HO-1 is a direct target of miR-218-5p

**Fig. 6:**
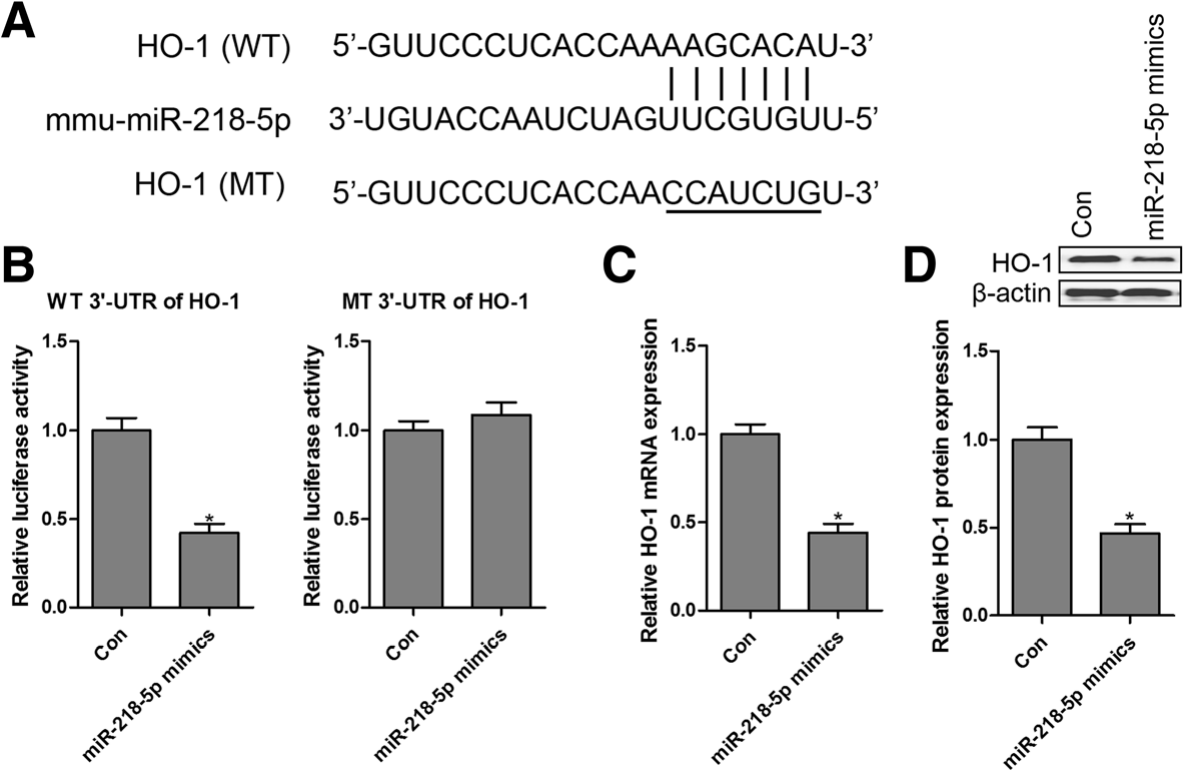
HO-1 is a direct target of miR-218-5p. The conserved binding sites between miR-218-5p and HO-1 were predicted using online software TargetScan and miRDB (**a**), and the luciferase activity assay was performed to validate HO-1 as a direct target of miR-218-5p (**b**). After transfection with miR-218-5p mimics into GMCs, the mRNA (**c**) and protein (**d**) expression levels of HO-1 were measured by RT-qPCR and western blotting, respectively. Values are expressed as mean ± SEM, *n* = 3 in each group. ^*^ *P* < 0.05 versus control group

The conserved binding sites between miR-218-5p and HO-1 were predicted using the online software TargetScan and miRDB, and the binding sites between miR-218-5p and HO-1 are shown in Fig. 6a. To confirm this prediction, a luciferase reporter assay was performed, and the results demonstrated that miR-218-5p mimics co-transfected with WT 3′-UTR of HO-1 significantly diminished the luciferase enzyme activity (Fig. 6b), while the luciferase enzyme activity showed no significant change in GMCs transfected with MT 3′-UTR of HO-1 (Fig. 6b). As shown in Fig. 6c and d, both mRNA and protein expression levels of HO-1 were dramatically reduced in GMCs transfected with miR-218-5p mimics compared with the control group. These findings showed that HO-1 was a direct target of miR-218-5p.

### Honokiol blocks LPS-induced cell growth inhibition and apoptosis by targeting miR-218-5p/HO-1

**Fig. 7:**
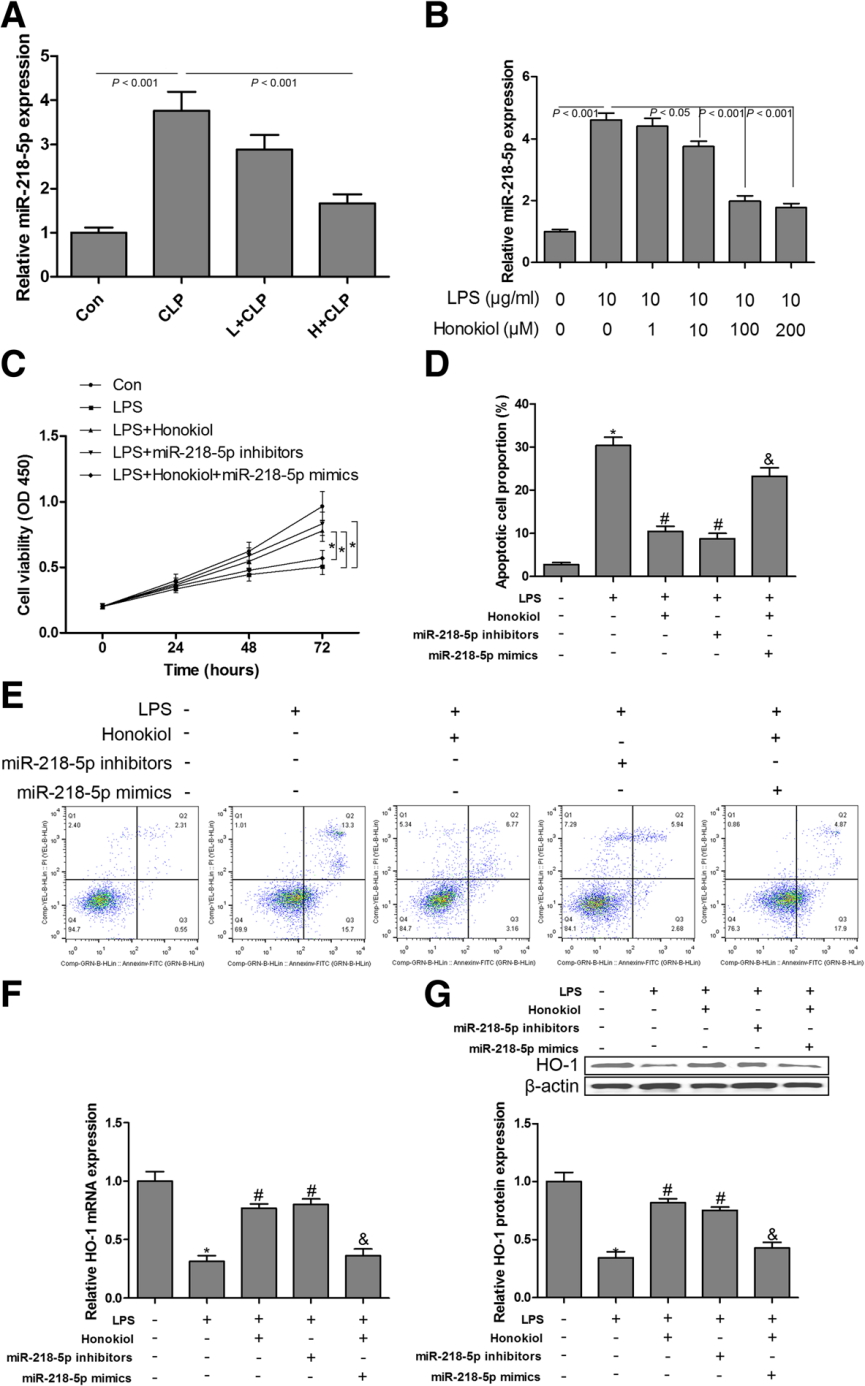
Honokiol blocks LPS-induced cell growth inhibition and apoptosis by targeting miR-218-5p/HO-1. The expression levels of miR-218-5p were measured using RT-qPCR assays in the kidney from septic mice with or without honokiol treatment (**a**) and in LPS-stimulated GMCs with or without honokiol treatment (**b**). GMCs were exposed to different conditions, cell viability was measured by CCK-8 assay (**c**); cell apoptosis was performed by flow cytometry (**d** and **e**); the mRNA (**f**) and protein (**g**) expression levels of HO-1 were measured by RT-qPCR and western blotting, respectively. Values are expressed as mean ± SEM, n = 3 in each group. ^*^ *P* < 0.05 versus control group; ^#^ *P* < 0.05 versus LPS treated group; ^&^ *P* < 0.05 versus honokiol+ LPS treated group

In honokiol-treated septic mice, sepsis-induced up-regulation of miR-218-5p in the kidney was markedly reversed by honokiol at the concentration of 10 mg/kg (Fig. 7a). We also performed in vitro experiments to investigate the protective effect of honokiol on LPS-stimulated GMCs. First, we found that honokiol inhibited LPS-induced up-regulation of miR-218-5p in GMCs in a concentration-dependent manner (Fig. 7b). LPS-induced cell growth inhibition (Fig. 7c) and apoptosis (Fig. 7d and e) in GMCs were reversed by both honokiol and miR-218-5p inhibitor treatment, while miR-218-5p mimics neutralized the protective effect of honokiol on LPS-stimulated GMC injuries (Fig. 7c, d and e). Furthermore, our results revealed that both honokiol and miR-218-5p inhibitors elevated the mRNA and protein expression of HO-1 in LPS-stimulated GMCs (Fig. 7f and g). However, overexpression of miR-218-5p reversed the up-regulation of HO-1 mRNA and protein expression by honokiol in LPS-stimulated GMCs (Fig. 7f and g). These findings indicated that miR-218-5p/HO-1 signaling was crucial for LPS-induced cell growth inhibition and apoptosis in GMCs, and that honokiol could suppress LPS-induced cell growth inhibition and apoptosis in GMCs by regulating miR-218-5p/HO-1 signaling.

## Discussion

In the present study, we demonstrated that honokiol had a beneficial effect on CLP-induced sepsis and significantly increased the survival in mice undergoing the CLP operation. Several inflammatory cytokines, such as TNF-α, IL-6 and IL-1β, were significantly inhibited in honokiol-treated septic mice as compared to the CLP single treatment group. Moreover, honokiol could markedly reverse CLP-induced AKI in septic mice. Importantly, the underlying mechanism revealed that HO-1 levels were significantly up-regulated and miR-218-5p levels were markedly down-regulated in honokiol-treated septic mice. Bioinformatics and experimental measurements revealed that HO-1 was a direct target of miR-218-5p. In vitro experiments showed that both honokiol and miR-218-5p inhibitors blocked LPS-induced cell growth inhibition and apoptosis in GMCs by increasing the expression of HO-1. These findings indicated that miR-218-5p/HO-1 signaling played an important role in sepsis-induced AKI and LPS-induced GMC dysfunction, and that honokiol could attenuate sepsis-induced AKI and LPS-induced GMC dysfunction by regulating miR-218-5p/HO-1 signaling.

Previous findings have demonstrated that honokiol is well tolerated by the host animal in therapeutically beneficial doses, and an acute toxicity study indicated that the LD_50_ of honokiol is 98 mg/kg administered intraperitoneally in mice [5]. The concentration of honokiol in our study was less than 10 mg/kg in septic mice, which ensured drug safety. Honokiol shows cytotoxicity and antimicrobial activity in *S. aureus* [26–28]. In our study, the bacterial counts in blood, kidney, liver and brain were significantly higher in the CLP group than the honokiol administration groups. These data suggest that honokiol exhibits strong bacterial killing ability in septic mice. Honokiol has been shown to ameliorate survival in a sepsis-induced acute lung injury mouse model and inhibit CLP- or LPS-induced oxidative stress and inflammation in lung tissues [5]. In CLP-induced septic rats, honokiol protected against sepsis-induced AKI through the inhibition of oxidative stress and inflammatory cytokine production and NF-κB signaling [11]. However, the precise mechanisms underlying sepsis-induced AKI still remain to be fully elucidated.

HO-1 is the rate-limiting enzyme in the degradation of heme to iron, carbon monoxide and biliverdin [18, 29]. A growing body of evidence suggests that HO-1 is involved in anti-inflammatory, anti-oxidant, anti-apoptotic, anti-proliferative and immunomodulatory effects that protect diverse organs against injury, including AKI [13]. A variety of rodent models have provided substantial evidence to support the role of HO-1 as a cytoprotective enzyme and its adaptive up-regulation after tissue injury [30]. In the AKI animal model, HO-1 mRNA is induced in the kidney as early as 3 to 6 h [31]. Genetically deficient HO-1 or inhibition of HO-1 activity with chemical drugs can accelerate kidney dysfunction and tubular injury [18], suggesting a protective role for HO-1 expression in kidney tissues. In a unilateral ureter obstruction mouse model, overexpressed HO-1 prevents renal interstitial inflammation and fibrosis by reducing macrophage infiltration and preventing the activation of Wnt/β-catenin signaling [32]. In the present study, we found that HO-1 was down-regulated in the kidneys following the CLP operation. Interestingly, down-regulated HO-1 was reversed in the kidneys of septic mice with honokiol administration. Our results indicated that HO-1 was involved in CLP-induced AKI in septic mice, and we also found that honokiol had a beneficial role in protecting against CLP-induced kidney injury by up-regulating HO-1 expression.

miR-218-5p as a post-transcriptional regulator repressed HO-1 expression in GMCs. Inhibition of miR-218-5p or honokiol administration blocked LPS-induced cell growth inhibition and apoptosis in GMCs by elevating HO-1 expression. Numerous studies have revealed that HO-1 exerts anti-inflammatory and anti-apoptotic effects on LPS-induced tissue damage and cell dysfunction [33, 34]. Up-regulation of HO-1 serves an anti-apoptotic role in LPS-treated hepatic cells, endothelial cells and cardiomyocytes [34–36]. Our results showed that miR-218-5p inhibitors modulated the up-regulation of HO-1 expression in GMCs, leading to the attenuation of LPS-mediated apoptosis. A recent study reported that miR-218 accelerated high glucose-induced podocyte apoptosis by down-regulating HO-1 expression [37]. These results suggested that up-regulation of miR-218-5p expression might be closely associated with AKI and cell apoptosis.

Mesangial cell apoptosis is implicated in septic renal injury, and LPS stimulates the release of inflammatory cytokines, such as TNF-α, IL-6 and IL-1β [38, 39]. LPS-stimulated mesangial cells are a convenient and reliable cell model to appraise the pathogenesis of sepsis-induced AKI in vitro. In addition, podocytes and proximal tubule epithelial cells are also recognized as cell models for AKI in vitro [40, 41]. In the present study, we selected only mesangial cells for research on the mechanism. However, we still believe that proximal tubule cells play a vital role in the pathogenesis of sepsis-induced AKI. Therefore, we intend to include proximal tubule cells in our future study.

In conclusion, our study demonstrated that honokiol could significantly ameliorate the pathological changes of kidney in septic mice induced by CLP and LPS-induced apoptosis in GMCs, and the underlying mechanism was mediated, at least partially, through the inhibition of miR-218-5p-mediated up-regulation of HO-1 expression.

## Additional file

Additional file 1: **Table S1.** Primers for RT-qPCR. (DOCX 14 kb)

## Abbreviations

3′-UTRs: 3′-untranslated regions
AKI: Acute kidney injury
CLP: Cecal ligation and puncture
GAPDH: Glyceraldehyde 3-phosphate dehydrogenase
GMCs: Glomerular mesangial cells
GSI: Glomerulosclerosis index
HO-1: Heme oxygenase-1
ICUs: Intensive care units
PAS: Periodic acid-Schiff
TIS: Tubulointerstitial score
