# Supplementary material for: Honokiol alleviates sepsis-induced acute kidney injury in mice by targeting the miR-218-5p/heme oxygenase-1 signaling pathway
Source: Cell Mol Biol Lett. 2019 Feb 22;24:15. doi: 10.1186/s11658-019-0142-4 (PMC6387556; doi:10.1186/s11658-019-0142-4)
Supplement: Supplementary file 1 — Table S1. Primers for RT-qPCR. (DOCX 14 kb) [file 11658_2019_142_MOESM1_ESM.docx]

**Additional file 1: Table S1. Primers for RT-qPCR**

| Gene | Forward primer (5′-3′) | Reverse primer (5′-3′) |
| --- | --- | --- |
| miR-7119-3p | ACACTCCAGCTGGGAAAAAACCGTTTC | TGGTGTCGTGGAGTCG |
| miR-377-3p | GGGAGGCAGTGTATTGTTA | CAGTGCGTGTCGTGGAGT |
| miR-7053-5p | ACACTCCAGCTGGGTGGGGAAACGGGCAGGC | TGGTGTCGTGGAGTCG |
| miR-3092-5p | ACACTCCAGCTGGGAGGGGAAAATGCCTTT | TGGTGTCGTGGAGTCG |
| miR-672-3p | TGAGGTTGGTGTACTGTGTGTGA | TGGTGTCGTGGAGTCG |
| miR-7231-5p | ACACTCCAGCTGGGTTGGGGAACACTGGGG | TGGTGTCGTGGAGTCG |
| miR-6975-5p | ACACTCCAGCTGGGGCTGGGGAGAAAGGGGT | TGGTGTCGTGGAGTCG |
| miR-7005-5p | ACACTCCAGCTGGGCCTGGGGATGGGAGGA | TGGTGTCGTGGAGTCG |
| miR-8108 | ACACTCCAGCTGGGTCTGGGGAGGAGCGTA | TGGTGTCGTGGAGTCG |
| miR-343 | ACACTCCAGCTGGGTCTCCCTTCATGTG | TGGTGTCGTGGAGTCG |
| miR-881-5p | ACACTCCAGCTGGGCAGAGAGATAACAGTC | TGGTGTCGTGGAGTCG |
| miR-6919-5p | ACACTCCAGCTGGGTAGGCCACTGGAGGTGG | TGGTGTCGTGGAGTCG |
| miR-7002-3p | ACACTCCAGCTGGGTTGTGCTTCCCCT | TGGTGTCGTGGAGTCG |
| miR-218-5p | CGAGTGCATTTGTGCTTGATCTA | TAATGGTCGAACGCCTAACGTC |
| miR-7026-3p | ACACTCCAGCTGGGTGTGCTTTCTGGTCTTG | TGGTGTCGTGGAGTCG |
| miR-134-5p | ATCTGTGACTGGTTGACCAGAGG | GTGCAGGGTCCGAGGT |
| miR-7020-3p | ACACTCCAGCTGGGAACCCCTCTCTTCTC | TGGTGTCGTGGAGTCG |
| U6 | CTCGCTTCGGCAGCACA | AACGCTTCACGAATTTGCGT |
